# Using foresight methodologies to tackle SARS-COV-2 related health impacts

**DOI:** 10.1093/eurpub/ckac129.199

**Published:** 2022-10-25

**Authors:** M Tijhuis, D Moye Holz, M Rodriguez, M Peyroteo, H Hilderink

**Affiliations:** 1 RIVM, Bilthoven, Netherlands; 2 NOVA, Lisbon, Portugal

## Abstract

**Background:**

A better understanding of possible future developments is essential for policy makers to anticipate and influence these trends. Public health foresight studies (PHFS) are tools to support this. The current health crisis makes clear that PHFS are necessary more than ever to target possible future impacts resulting from SARS-COV-2 induced changes in e.g. regular health care services, lifestyle and socio-economic developments. To support European countries in doing their own foresight studies, PHIRI aims to strengthen foresight capacity.

**Methods:**

PHIRI follows a 4-step approach: 1. Making an inventory of current PHFS capacities and capacity needs across European countries using desk research and an online survey; 2. Providing PHFS capacity building via a training course and workshops; 3. Supporting the development of public health scenarios for the short term (0-5 years) and longer-term (5-20 years); 4. Supporting the identification of promising policy strategies, using policy dialogues.

**Results:**

The online survey was completed by participants of 21 countries and shed light on existing national PHFS and needs for capacity building. It also provided a basis for the development of a professional network on PHFS within the project. A PHFS capacity building course was developed, including videos posted on the PHIRI website and a template structuring the different foresight elements. Around 15 researchers undertook their own PHFS, covering a wide range of public health topics, using the material provided during the course. In addition, a compact guide was developed and provided, explaining the different foresight elements. Based on these studies, common challenges and promising policies are identified.

**Conclusions:**

Foresight in public health is gaining more and more interest, especially now in these times of crisis. PHIRI provides more insight in the wider public health impacts of the SARS-CoV-2 pandemic and in translating this into policy options.

